# Corrigendum: Cortico-striatal spike-timing dependent plasticity after activation of subcortical pathways

**DOI:** 10.3389/fnsyn.2015.00013

**Published:** 2015-08-31

**Authors:** Jan M. Schulz, Peter Redgrave, John N. J. Reynolds

**Affiliations:** ^1^Department of Biomedicine, University of Basel Basel, Switzerland; ^2^Department of Psychology, University of Sheffield, Western Bank Sheffield, UK; ^3^Department of Anatomy, School of Medical Sciences, University of Otago Dunedin, New Zealand

**Keywords:** STDP, striatum, superior colliculus, spiny projection neuron, HFS, *in vivo*, intracellular, dopamine

The arrangement of the panels in Figure 4 of the article by Schulz et al. (2010) is incorrect. The order of the panels A and B in Figure 4 was erroneously reversed, so that the graphs do not match the figure legend and the references in the text. Here, we provide the original figure with the correct panel arrangement.

**Figure 4:**
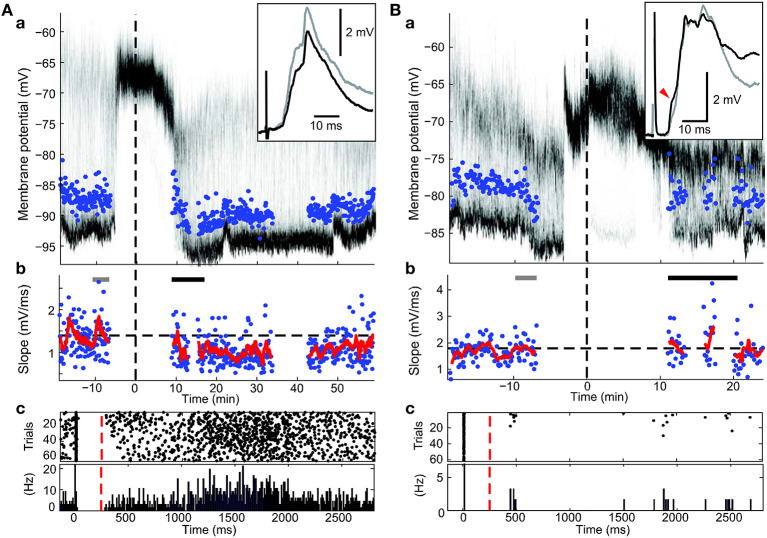
**Effects of post-light spike rate on cortico-striatal plasticity in experiments involving pre-post pairing**. (a) Time-resolved membrane potential distribution. Gray-scale indicates the probability for the neuron to be at respective membrane potential (y-axis); black depicts a high probability, white a low probability. Time on the x-axis is given in relation to BIC ejection (dotted line at 0). Maximum amplitude of PSPs is indicated. (b) Maximal slopes of PSP. Dashed line indicates mean PSP slopes before at baseline. Running average of 9 consecutive values is indicated (red trace). PSP traces (top right inset in a) are mean PSPs recorded in the time indicated by the gray and black bars, respectively. (c) Raster plot and peri-stimulus time histogram of spikes during the pairing protocol. Cortical stimulus was applied at 0, the intracellular current pulse at approximately 10 ms, and light at 250 ms (red dashed line). **(A)** Example recording showing depression (*p* < 0.001; Wilcoxon rank sum test). Note the high spike rate post-light. **(B)** Example recording showing a selective increase in a PSP component (red arrow head; *p* < 0.05; Wilcoxon rank sum test) without a significant change of the overall PSP slope.

## Conflict of interest statement

The authors declare that the research was conducted in the absence of any commercial or financial relationships that could be construed as a potential conflict of interest.

